# Employment pattern and depression for middle-aged and older natives and immigrants in the United States

**DOI:** 10.1093/geroni/igaf122.121

**Published:** 2025-12-31

**Authors:** Yingyue Jiang

**Affiliations:** Brown University, Providence, Rhode Island, United States

## Abstract

Stable full-time employment and voluntary retirement are important to the well-being of older adults, in terms of economic status and health. This study examines the employment and retirement pattern of middle-aged adults to older adults, as well as these patterns’ association with depression. This study focuses on differences between natives and immigrants in the United States, using data from the 1992-1998 Health and Retirement Study (HRS) of 4,706 natives and 513 immigrants who were aged 50 to 65 at the baseline in the United States. Sequence analysis and multivariate analysis were employed to identify key employment and retirement patterns. Findings show that natives typically exhibit greater stability in full-time employment while immigrants show more variation in transition patterns. Among respondents who have full-time employment before exiting the labor market, natives are more likely to maintain their original status and exit the labor market later. Among respondents who indicated retired, there is no significant difference in the type of retirement observed between natives and immigrants. Immigrants have higher depression scores in both the first wave and last wave compared to natives. Early exit from the labor market and job instability (part-time employment and unemployment) are associated with higher levels of depression, with a greater impact observed among immigrants. The nonsignificant effect of years in the U.S. challenges the assimilation hypothesis and underscores persistent health inequalities among immigrants. The findings highlight the importance of job stability and voluntary retirement in supporting mental health and reducing social inequalities among middle-aged to older adults.

